# The acceptability, feasibility, and preliminary efficacy of a supported online self-help treatment program for binge-eating disorder

**DOI:** 10.3389/fpsyt.2023.1229261

**Published:** 2023-10-04

**Authors:** Sean Rom, Jane Miskovic-Wheatley, Sarah Barakat, Phillip Aouad, Marcellinus Kim, Matthew Fuller-Tyszkiewicz, Sarah Maguire

**Affiliations:** ^1^Faculty of Health, School of Psychology, Deakin University, Geelong, VIC, Australia; ^2^InsideOut Institute, The University of Sydney and Sydney Local Health District, Sydney, NSW, Australia; ^3^Sydney Local Health District, Sydney, NSW, Australia

**Keywords:** binge-eating disorder, eating disorders, binge-eating, eTherapy, online therapy, digital therapy, cognitive behavioural therapy, self-help

## Abstract

**Introduction:**

Studies in transdiagnostic eating disorder (ED) samples suggest supported online self-help programs (eTherapies) are effective and may improve access to treatment; however, their evaluation in those with binge-eating disorder (BED) is limited. Given BED’s high prevalence and low levels of treatment uptake, further eTherapy evaluation is needed to broaden access to effective, evidence-based treatment options. The aim of this study was to investigate the acceptability, feasibility, and preliminary efficacy of a supported eTherapy for those with BED or subthreshold BED, and to examine symptom change across the duration of therapy.

**Method:**

Nineteen women with BED completed a supported, 10-session Cognitive Behavioural Therapy-based eTherapy in an uncontrolled, pre-post, and 3 months follow up intervention study. Key outcomes were assessed by the Eating Disorder Examination Questionnaire (EDE-Q): objective binge episode (OBE) frequency and ED psychopathology. Feasibility was evaluated via program adherence and dropout, whilst acceptability was assessed through participant feedback post-treatment. Weekly symptom change (ED psychopathology) during treatment was assessed by the Eating Disorder Examination - Questionnaire Short (EDE-QS).

**Results:**

Generalised estimating equations showed statistically and clinically significant reductions in OBEs and ED psychopathology (large effects) post-treatment, with these decreases maintained at follow up. Across weekly assessment, a marked slowing in the rate of change in ED psychopathology was observed after four sessions of the program. Program feasibility was high (i.e., 84% of content completed), as was program acceptability (i.e., 93% of participants expressed high levels of satisfaction).

**Discussion:**

These results support the acceptability, feasibility, and preliminary efficacy of a supported eTherapy program for those with BED and suggest the variability of symptom change across the duration of therapy. Future research should further investigate findings in an adequately powered randomised controlled trial.

## 1. Introduction

Binge-eating disorder (BED) is characterised by the consumption of an objectively large amount of food at least once per week in the previous 3 months, with a sense of being incapable of controlling the eating (1). Although binge-eating is a shared feature of other eating disorders (ED), BED’s sociodemographic profile is unique (2). Specifically, BED has a more equal gender split, less severe dietary restraint, non-compulsory over-evaluation of weight and shape, and a higher proportion of individuals at a higher weight compared with other EDs (2, 3). BED is also the most common ED, with a conservative estimated prevalence of 1.9% across the lifespan (4). Contrary to its common misconception as a “mild” illness (5), evidence suggests those with BED experience distressing symptom episodes and have the longest duration of untreated illness amongst EDs (6, 7). Furthermore, BED often features complex co-occurrences with obesity, depression, anxiety, substance abuse, personality disorders and suicidality, and ultimately carries an increased risk of serious long-term health consequences, such as Type 2 diabetes (8, 9).

Despite the potential for protracted and distressing symptomatology in BED, there are evidence-based treatments. First-line treatment comprises 20-h or more of individual face-to-face Cognitive Behavioural Therapy (CBT), targeting the maladaptive behaviours, thoughts, and feelings that maintain the ED (10). However, shortages of appropriately skilled professionals fuel an unmet demand (11) and this is magnified in rural or remote populations that often lack access to specialised care (12). In addition, poor recognition of non-underweight ED presentations impacts detection in the health system (13, 14) and those who are identified encounter high costs for individual face-to-face therapy (15) or commonly experience treatment-seeking hesitancy due to self-stigma, waiting lists, and previous negative treatment experiences (16, 17). The confluence of these factors contributes to inadequate treatment uptake, with a review of studies across high-income countries reporting those with BED access care at rates as low as 10% (18). Treatment alternatives that emphasise scalability, cost-effectiveness, anonymity, autonomy, and convenience are, therefore, urgently needed to increase help-seeking (11).

Guided self-help programs have emerged as promising lower-intensity alternatives to face-to-face treatment delivery (19). These programs provide structured, evidence-based interventions that individuals complete themselves, with varying levels of support provided by a clinician or an appropriately trained support person (20). Their provision in cases of mild to moderate non-underweight EDs, such as BED or bulimia nervosa, is increasingly recommended within a stepped-care model in which psychological treatment intensity is scaled according to illness acuity (21). CBT comprises the therapeutic approach for a majority of these guided self-help interventions: a meta-analysis of transdiagnostic, binge-eating targeted eTherapies (i.e., digitally delivered evidence-based therapy) reported CBT-based interventions in 79% of included studies (22). eTherapies themselves have emerged as a preferred guided self-help format, their online delivery addressing issues of accessibility, scalability, cost, and privacy (23, 24), whilst presenting unique opportunities for innovation, interactivity, and engagement via multimedia content and other design features (22). Moreover, their efficacy in reducing ED and binge-eating symptomatology in transdiagnostic ED samples has growing evidence across multiple meta-analyses, with small to medium average effect sizes reported (22, 25–27).

Despite their promise, eTherapy evaluation in BED-specific samples is limited (26). A meta-analysis of BED eTherapy programs found moderate pooled reductions in objective binge episode frequency (*d* = −0.77) and ED psychopathology (*d* = −0.71) across three studies (28–30). Although these initial results appeared promising, the authors concluded that BED eTherapies had an insufficient quantity of evidence to support their efficacy compared with face-to-face CBT (31). The three included studies evaluated eTherapy interventions that ranged between 11 and 21 sessions. All studies used superseded DSM-IV diagnostic criteria for BED diagnosis in their samples; two employed clinical psychologists in the support clinician role (28, 29), with only one study utilising other health professionals (30). Given the long waitlists for access to services, and the goal of eTherapy programs to be accessible and scalable treatments, the lack of program evaluation with other appropriate support professionals limits the generalisability of these findings. Overall, the small number of studies evaluating eTherapy programs in those with BED is problematic given BED’s status as the most common eating disorder and the identified need for more accessible, evidence-based treatment alternatives, like eTherapies, to address low rates of treatment uptake in this clinical group. Clearly, further evaluation of eTherapies in purely BED samples is urgently needed (31).

The Supported Self-Help Binge-Eating eTherapy (SSH-BEeT) Program was developed as a low-intensity online self-help program for the treatment of binge-eating symptomatology and ED psychopathology in those with BED. A preliminary evaluation of the program found promising reductions in key ED symptomatology in individuals with BED who completed the first four sessions of treatment, which comprise the Brief Supported Self-Help BEeT Program (Brief SSH BEeT) (32). Given Brief SSH-BEeT predominantly encompasses behavioural interventions (i.e., establishing regular eating patterns, self-monitoring of food-intake, etc.) that are commonly employed in the early sessions of a CBT program for BED, it was suggested that these behavioural components may drive the early and substantial symptom change often seen in the first 4 weeks of CBT treatment for BED (33, 34). This aligns with emerging evidence in CBT-based programs for EDs that suggest shorter duration programs may be as effective at reducing key ED psychopathology (35).

Given uncertainty around the minimum required dose for meaningful clinical change, it was pertinent to explore further symptom change in this same sample, who were provided six more sessions of eTherapy content. An exploration of weekly symptom change across the 10 weekly sessions would provide further information regarding trends and patterns of change, i.e., whether the promising reductions in ED psychopathology observed after four sessions were maintained, enhanced, or slowed with further sessions. Furthermore, an evaluation of the 10-session program at pre-post with 3 months follow up would provide preliminary efficacy data on the full program (which introduces key cognitive strategies in later sessions) and the durability of any symptom change for individuals with BED.

This study aimed to investigate the acceptability, feasibility, and preliminary efficacy of the 10-session supported eTherapy intervention for people with BED or subthreshold BED, in addition to examining symptom change across the duration of therapy. It was hypothesised that participants with BED or subthreshold BED, who completed the 10-session Supported Self-Help Binge-Eating eTherapy (SSH-BEeT) program, would have a significantly reduced frequency of objective binge episodes and reduced overall ED psychopathology at post-treatment and 3 months follow up, compared with baseline assessment. The weekly rate of change in ED psychopathology was also explored. Feasibility was evaluated via adherence to program content, dropout, and preliminary efficacy findings, whilst acceptability was assessed from feedback provided by participants at post-treatment assessment.

## 2. Materials and methods

### 2.1. Design

This uncontrolled study presents results from a supported 10-session eTherapy program (SSH-BEeT), extending findings from a previous evaluation after four-sessions of treatment (SSH Brief BEeT) in the same cohort (32). A repeated measures design was employed across three timepoints, with the within group variable the time of intervention exposure, i.e., pre-intervention, post-intervention, and three-month follow up. The study setting was predominantly digital, i.e., via the eTherapy platform and secure video conferencing.

### 2.2. Participants

**Figure 1:**
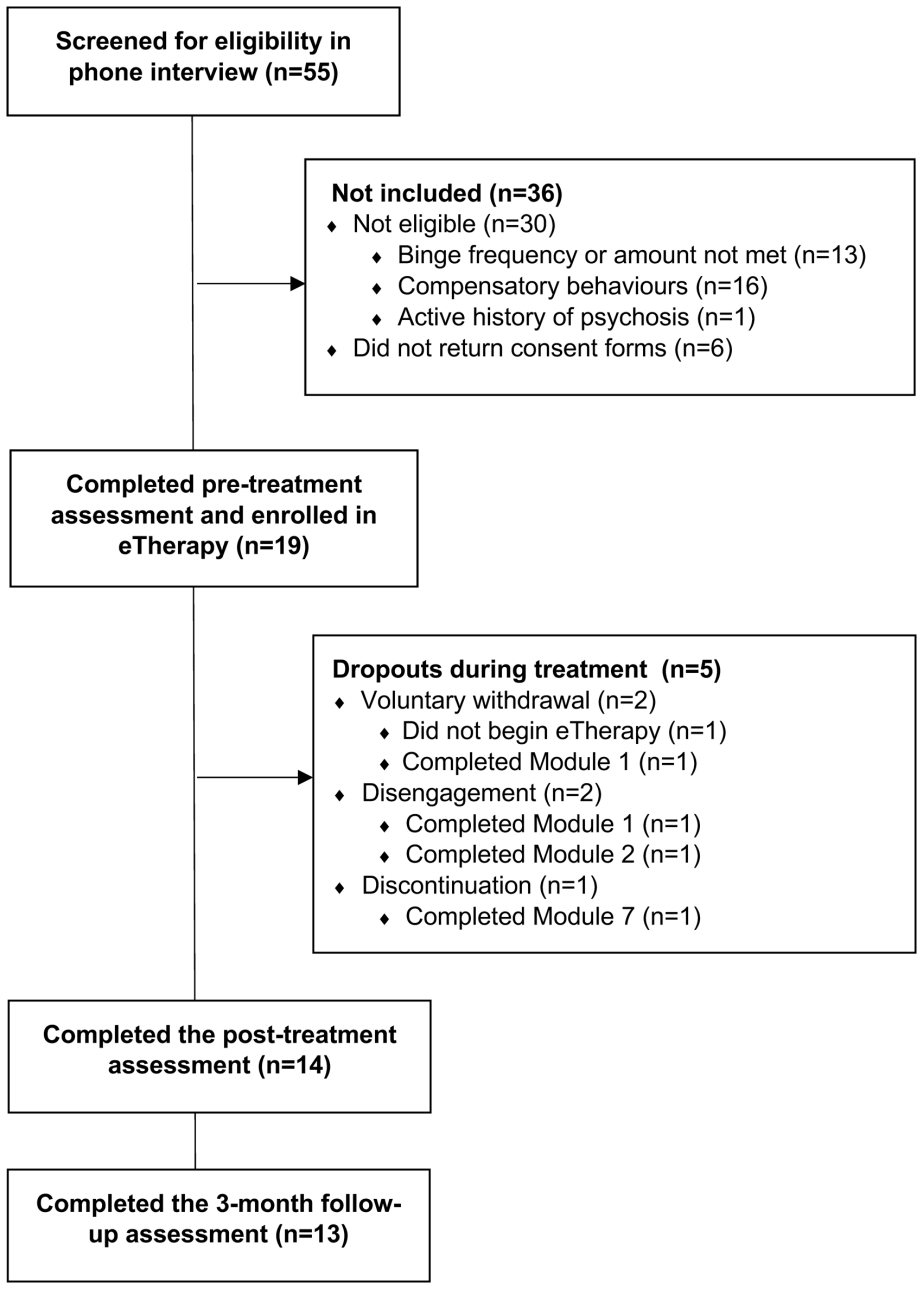
Study participant flowchart. Dropouts are defined as participants who did not complete the post-treatment questionnaire. Program disengagement was defined by participants who missed three or more consecutive support sessions.

Participants were all English-speaking and recruited via online advertising (i.e., Facebook advertising and the HealthMatch clinical trial registry) from the Australian community (see CONSORT flow diagram in Figure 1) between March and December 2021. Fifty-five women expressed interest via an online form, with 19 women entering the study following a 20-min screening phone call with a research assistant using self-designed questions (36). The research assistant was trained by a clinical psychologist and received regular supervision. Interview questions evaluated the severity, frequency, and duration of binge-eating symptomatology as per the DSM-5 criteria for BED, in addition to general mental health history (i.e., major psychiatric history, including suicidality, history of psychosis, current medications, etc.), and other study criteria such as internet and video devise access, and current weight and height. To be eligible, participants were required to meet the DSM-5 criteria for BED or Other Specified Feeding or Eating Disorder with BED behaviours (subthreshold BED), i.e., experienced a minimum of one or more weekly objective binge episode in the preceding 2 months (1). Additional criteria included an age of 16 years or older, a BMI equal to or greater than 20, and access to both internet and a digital device with video camera.

Participants were excluded if they were currently receiving psychological treatment for BED, were pregnant or breast-feeding, using stimulant medication, were non-proficient English speakers, and were psychiatrically (i.e., disclosed a history of psychosis or were actively suicidal) or medically unstable (based on assessment by a GP).

### 2.3. Materials

#### 2.3.1. Online eTherapy intervention

Supported Self-Help Binge-Eating eTherapy (SSH-BEeT) encompasses 10 online sessions, which deliver a low-intensity, CBT-based intervention for BED. As described in Table 1, the SSH-BEeT program focuses on key principles, including behavioural techniques to establish a regular eating pattern (i.e., eating every 3 h) and exposure techniques such as weekly weighing (i.e., to promote habituation to the distress caused by the concept of weight and weighing or contain/reduce frequent weighing), feared food experimentation, and exercises to address body checking and avoidance. The first four sessions (i.e., Brief SSH-BEeT) are predominantly dedicated to behavioural interventions (i.e., establishing regular eating, weekly weighing), with cognitive, emotional-regulation, and further behavioural interventions introduced from module five onwards. Each weekly module takes approximately one-hour to complete and is presented via diverse multimedia content, with a pre-recorded therapist delivering core treatment principles. The program is highly interactive, with experiential digital exercises and in-built self-monitoring tools, such as food and behaviour monitoring, food planning, thought challenging, and exposure tools. In addition, the program prompts self-reflection of behavioural change and provides automated feedback on improvements in key symptomatology (i.e., objective binge-episodes, overeating, etc.).

**Table 1:** eTherapy weekly module content.

Module	Key principles
Formulation and monitoring	CBT psychoeducation and description of the binge-eating cycle. Introduction and justification for self-monitoring of eating, including digital food diary use to log daily food and plan future meals. Introduction of weekly weighing.
Eating regularly and planning	Personalised case formulation and psychoeducation regarding food restriction and starvation. Introduction of the three-hour rule (regular/structured eating every 3 h) and psychoeducation regarding normal eating.
Addressing binges	Strategies and skills to address binge-eating and the urge to binge. Overview of the role of triggers in the binge-eating cycle.
Problem solving and motivation	Introduction of a problem-solving framework, an introduction to feelings and their role in the CBT model, and motivational strategies.
Understanding and noticing thoughts and feelings	Introduction of an emotional regulation framework to assist with negative emotions. Self-monitoring of unhelpful thoughts using digital tool.
Coping with thoughts and feelings	Introduction to thought challenging.
Exposure challenges: Feared foods and food rules	Psychoeducation with regards to “feared foods” and “food rules.” Development of an exposure hierarchy to challenge feared foods and food rules using digital Exposure Tools.
Exposure challenges: Body image	Psychoeducation regarding body image and the development of personalised exposure exercises to challenge body checking and/or avoidance behaviours. Introduction to “Urge Surfing.”
Self-compassion and identifying values and strengths	Identifying strategies to encourage greater acceptance and self-compassion, including reflection of strengths and values separate from eating, weight, and shape.
Review and relapse prevention	Introduction of relapse prevention strategies in a recovery-based framework. A review of symptom-change and a discussion regarding possible engagement with further treatment and supports.

This table was originally published in Rom and colleagues (32) and has been modified to include additional information about eTherapy modules 5 to 10. This table is part of an open access article under the terms of the Creative Commons Attribution-NonCommercial-NoDerivs License, which permits use and distribution in any medium, provided the original work is properly cited, the use is non-commercial, and no modifications or adaptations are made.

#### 2.3.2. Support sessions

After completing the prescribed weekly module of eTherapy content, participants also attended a weekly guided session (30-min) with a clinician via videoconferencing. These 10 sessions provided additional support as participants completed the eTherapy. The support clinician monitored participant’s completion of program content, including self-monitoring of food intake, thoughts, feelings, etc., and were guided by manualised instructions and standardised questions to explore the content and module tasks from the previous week. Clinicians were research assistants with various qualifications in psychology and dietetics. All support clinicians completed online training and attended regular clinical supervision with a psychologist trained in this approach to ensure adherence to the treatment protocol. Therapeutic contact with participants is outlined in Table 2.

**Table 2:** Therapeutic Contact Frequency.

Form of contact	Frequency
Personalised contact	1 × Introductory session (1 h); 10 × weekly guided support sessions (30 min).^†^
Automatised contact	Daily SMS reminders for food logging at 9 am, with an additional reminder at 6 pm if no logging for 2 days; Email notification when upcoming eTherapy module is unlocked.
Other	GP medical assessment before program onboarding; continual GP medical monitoring across trial duration as advised by GP.

† Guided session duration was tracked by the support clinician after completion of each session to evaluate and monitor fidelity to the treatment protocol.This table was originally published in Rom and colleagues (32) and is part of an open access article under the terms of the Creative Commons Attribution-NonCommercial-NoDerivs License, which permits use and distribution in any medium, provided the original work is properly cited, the use is non-commercial, and no modifications or adaptations are made.

#### 2.3.3. Measures

**Figure 2:**
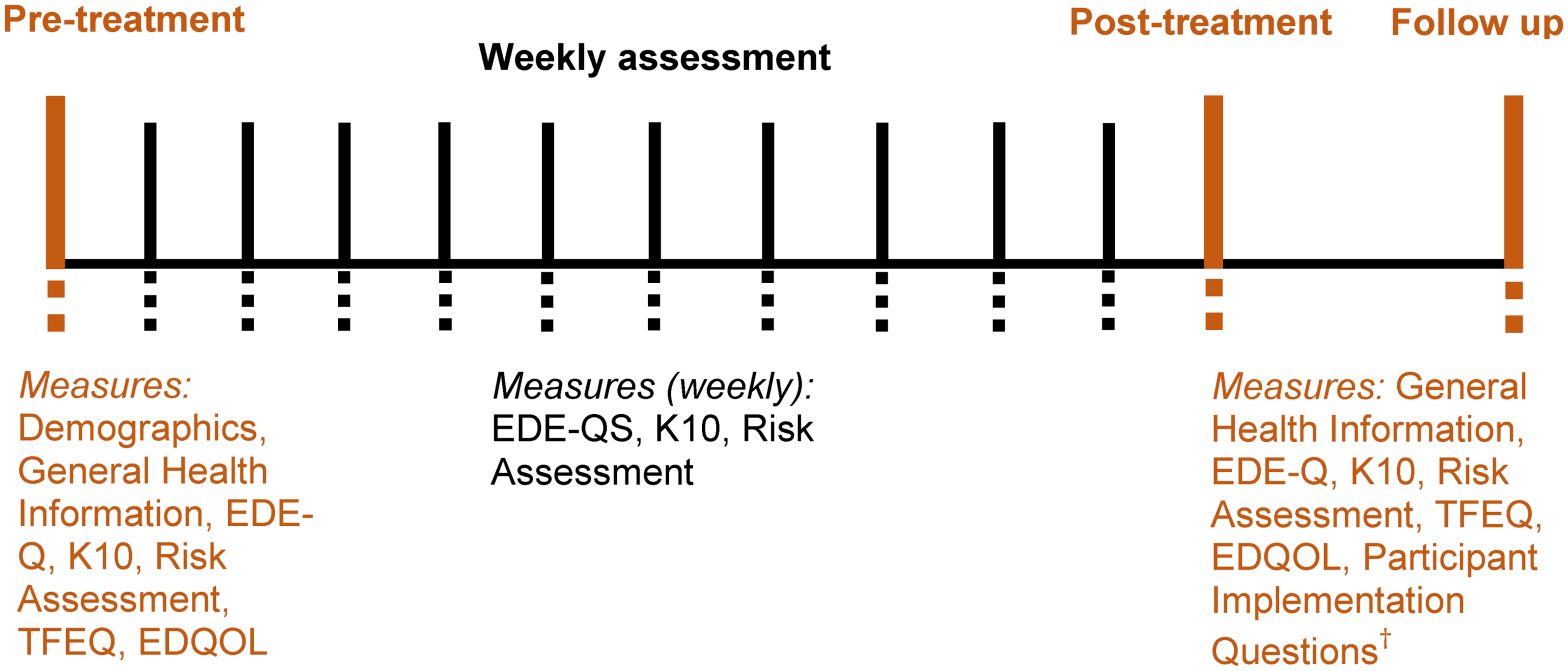
Timing and sequence of assessment. Weekly assessment during completion of the eTherapy, before completing each weekly module of content. Post-treatment assessment was approximately one week after completion of the program. Follow up occurred three months after post-treatment. EDE-Q = eating disorder examination - questionnaire; EDE-QS = eating disorder examination - questionnaire short; K10 = Kessler psychological distress scale; TFEQ = the three factor eating questionnaire; EDQOL = eating disorder quality of life questionnaire. ^†^Provided only in post-treatment assessment.

Measures were digitally delivered self-report questionnaires, with assessment timepoints outlined in Figure 2.

##### 2.3.3.1. Eating disorder psychopathology

The Eating Disorder Examination-Questionnaire (EDE-Q) (37) was employed to measure the study’s primary outcomes: objective binge episode (OBE) frequency and ED psychopathology via the EDE-Q global score. This psychometric measure assesses self-reported ED symptomatology, including frequency of binge-eating (i.e., OBE frequency) and other ED behaviours and attitudinal-related ED psychopathology (i.e., thoughts around shape, weight, eating, etc.) in the previous 28 days across 30-items and four subscales (i.e., restraint, eating concern, shape concern, and weight concern), with higher sores indicative of more severe ED psychopathology. The EDE-Q upholds good reliability (α = 0.90) (38) and validity (39) and has been widely utilised to assess those with BED (40).

The Three Factor Eating Questionnaire (TFEQ) (41) measures the behavioural and cognitive aspects of eating across 51-items in the previous 28 days. The measure comprises three subscales, cognitive restraint of eating (α = 0.93), disinhibition (α = 0.91), and hunger (α = 0.85), which uphold good reliability and validity (41).

The Eating Disorder Examination – Questionnaire Short (EDE-QS) (42) is a psychometric measure derived from the EDE-Q to evaluate ED symptomatology in the preceding 7 days across 12-items, with a higher total score indicative of more severe ED psychopathology. This measure was employed in weekly questionnaires and has good reliability (α = 0.91) (42) and validity (43).

##### 2.3.3.2. Psychological distress

The Kessler Psychological Distress Scale (K10) (44) is a psychometric measure of psychological distress evaluated across 10 items, with higher scores indicative of increased negative emotionality in the previous seven (employed in weekly questionnaires) and 28 days (employed in pre-post treatment and follow-up questionnaires). This measure has good reliability (α = 0.93) (44) and validity (45).

##### 2.3.3.3. Illness-related quality of life

The Eating Disorder Quality of Life Questionnaire (EDQOL) (46)assesses ED-specific quality of life across 25-items and four subscales (i.e., psychological, physical/cognitive, work/school, and financial) in the preceding 28 days, with higher scores indicative of lower quality of life. The EDQOL has good reliability (α = 0.94) and validity (46).

#### 2.3.4. Demographic and general health information

Questions comprised basic socio-demographic information, including age, gender, occupation, ethnicity, education, and residential setting. In addition, there were general health questions, such as the presence of secondary physical or mental health concerns.

##### 2.3.4.1. Risk assessment

A self-designed questionnaire (36) assessed the severity of participant’s suicidal and self-harm behaviours across the previous 28 days to 12 months (employed in pre-post treatment and follow-up questionnaires) and the previous 7 days (utilised in weekly questionnaires).

##### 2.3.4.2. Feasibility

Measured by program dropout, preliminary efficacy findings (i.e., pre-post and follow up treatment evaluation of primary outcomes), and program adherence indices (based on data extracted from the eTherapy program). Adherences indices include percentage completion of content (out of a possible 10 eTherapy modules), attendance at support sessions (out of a possible 10 sessions), the total number of program logins, the number of self-monitoring entries (i.e., food, thought, behaviour, food planning, goal setting, exposure challenges), and the average amount of self-monitoring entries per login.

##### 2.3.4.3. Acceptability

Evaluated by a self-designed questionnaire of 14-items (36), which assessed the perceived usefulness of the intervention for participants, including the skills and components of the eTherapy, or other factors, that helped and hindered their ability to complete the program.

##### 2.3.4.4. Adverse effects

This was evaluated weekly (during program completion) via risk assessment (i.e., suicidal ideation and self-harm) and psychological distress/negative affect (i.e., The Kessler Psychological Distress Scale) questionnaires and monitored qualitatively in weekly telehealth support sessions with support clinicians. In addition, this was assessed via feedback provided by participants in the acceptability questionnaire post-treatment (i.e., the participant’s perspective of the quality of treatment), or other communications to the research team.

### 2.4. Procedure

Those eligible for participation were required to visit their GP to confirm their medical stability. The GP was provided with information on the study, in addition to recommendations on possible areas of assessment (i.e., weight, pulse, blood pressure, fasting glucose, electrolytes, renal function, etc.). The GP was then required to sign a medical clearance form and agree to medically monitor the participant during the study period. Participants were then sent study information and consent forms, with those aged 16–17 years old required to provide additional parental consent. Participants also consented to researchers contacting their GP if psychiatric risk emerged. After receiving these signed documents, participants were then sent an online pre-treatment assessment. The voluntary nature of participation was reiterated throughout all stages of the trial.

Prior to beginning each weekly eTherapy module, participants also completed an online weekly assessment comprising the EDE-QS, K10, and Risk Assessment questions (see Figure 2). In addition to completing weekly eTherapy content, participants self-monitored via the digital diary and attended a scheduled weekly telehealth support session, after which their support clinician unlocked the next weekly eTherapy module for completion. Participants who did not attend their scheduled support sessions were contacted by researchers to prompt re-engagement; if three or more consecutive support sessions were missed without further contact, the participant was considered disengaged from the study. The duration of SSH-BEeT is 10-weeks; however, including pre-and post-assessment, was approximately 12-weeks. Follow up assessment occurred approximately 12-weeks after post-assessment.

Risk associated with participation was low; however, given the clinical population, participant safety was evaluated on initial screening and safety planning was conducted if a history of suicidality or self-harm was reported. In addition, weekly risk assessment questions were administered to all participants. If suicidal ideation or self-harm were reported via online measures, an automatic email was sent to researchers prompting them to complete a phone call with the participant to confirm their safety. In such cases, the participant’s GP was also contacted to determine whether the participant could safely continue in the program.

All participant data were stored on an online database via a secure, firewall protected website as per the University of Sydney data management guidelines. Access required a login and password provided only to authorised members of the research team. Ethical approval for this study has been provided by the Sydney Local Health District RPA Research Ethics and Governance Office (Ethics Approval Number: X18-0486 and 2019/ETH12146) and the Deakin University Human Research Ethics Committee (Ethics Approval Number: 2021-145). Furthermore, the study has been registered with the Australia New Zealand Clinical Trials Registry (ANZCTR Registration Number: ACTRN12621001612808).

### 2.5. Data analysis

Data were cleaned and all analyses run via SPSS (v.26) and Stata (v.18). Initial exploratory analyses were conducted with descriptive statistics to summarise the results with measures of central tendency and dispersion. Greater than 5% of missing data were considered significant and analysed with appropriate statistical models based on missingness assumptions that fit the data (missing at random vs. missing not at random) and with intention to treat principles. Generalised estimating equations (GEE) with multiple imputation were employed to evaluate change in primary (i.e., objective binge episode frequency and ED psychopathology) and secondary outcomes after initial analyses were completed with GLMM and these models were found to be invalid as within subject variance in the model was near zero, thereby violating the assumption that this is correlated data. Given the three timepoints (i.e., pre-treatment, post-treatment, and three-month follow up), GEE with an exchangeable covariance structure was identified as an appropriate approach as it requires only one covariance parameter to specify, thus an exchangeable covariance structure was valid. This mitigated the usual challenges with GEE of choosing the most appropriate covariance structure. Clinically relevant covariates of age, BMI, K10 total score, and EDE-Q global score were included in the models to control for their possibly confounding effects on outcomes. Analyses were conducted across all three timepoints (i.e., pre-treatment, post-treatment, and three-month follow up), and evaluating pre-to post-treatment, and post-treatment to three-month follow-up. The primary endpoint to establish preliminary efficacy across outcomes was post-treatment, with the follow up timepoint employed to establish the maintenance or durability of any change.

Clinically significant change was evaluated using the following established metrics. With GEE, the beta coefficients output from the model has direct clinical interpretation and therefore equates to an effect size. In addition, Hedge’s *g* with small sample correction was calculated for primary outcomes for additional confirmation. Abstinence from binge-eating was defined as zero OBEs in the previous month, recovery from binge-eating disorder was defined as <4 OBEs in the previous month as per Wagner and colleague’s approach (29), and ‘good outcome’ was defined as an EDE-Q global score at post-treatment and follow up <1 SD above the community mean (<2.77) in Australian adult females (47), consistent with the method employed in CBT-ED studies (35, 48–50).

## 3. Results

### 3.1. Participant characteristics

Participants (*N* = 19) were female identifying, aged 19.55 to 53.81 (*M* = 37.69, *SD* = 10.52), had a body mass index (BMI) of 20.30 to 44.80 (*M* = 31.13, *SD* = 9.86), and an illness duration of 2.02 to 45.81 years (*M* = 21.38, *SD* = 13.65). Although the study was open to participants who had experienced at least one weekly objective binge episode in the previous 2 months (i.e., subthreshold BED/Other Specified Feeding or Eating Disorder with BED behaviours), all participants (*N* = 19) reported this frequency over the previous 3 months or more, consistent with the DSM-5 criteria for BED (1). With regards to symptom severity, 95% (*n* = 18) of participants fell within the mild to moderate severity range and 5% (*n* = 1) were in the severe range. A majority of participants (74%, *n* = 14) reported a secondary mental health concern alongside eating and weight issues, with anxiety the most common (47%, *n* = 9). Further socio-demographic participant characteristics can be found in Table 3.

**Table 3:** Participant characteristics at baseline (*N* = 19).

Baseline characteristic	Sample
Employment, *n* (%)	
Full-time	10 (52.63)
Part-time	7 (36.84)
Unemployed or student	2 (10.53)
Education level, *n* (%)	
High school	1 (5.26)
Some university or tertiary study	3 (15.79)
Bachelor’s degree or post-graduate study	15 (78.95)
Annual gross income in Australian dollars, *n* (%)	
5,000 to 9,999	2 (10.53)
20,000 to 39,999	4 (21.05)
40,000 to 69,999	5 (26.32)
70,000 to 119,999	4 (21.05)
120,000 to 149,999	1 (5.26)
150,000 or more	3 (15.79)
Cultural background^a^, *n* (%)	
Australian	12 (63.16)
Aboriginal or Torres Strait Islander	1 (5.26)
New Zealand	2 (10.53)
South American	1 (5.26)
Multiple races	3 (15.79)
Setting of residence, *n* (%)	
Metropolitan	13 (68.42)
Regional	6 (31.58)
Primary mental health concerns, *n* (%)	
Eating/weight issues	14 (73.68)
Anxiety	2 (10.53)
Depression	2 (10.53)
Other	1 (5.26)
Secondary mental health concerns, *n* (%)	
Anxiety	9 (47.37)
Stress	3 (15.79)
Depression	2 (10.53)
Eating/weight issues	3 (15.79)
None	2 (10.53)
Other mental health services currently accessed^b^, *n* (%)	
Psychologist	8 (42.11)
Psychiatrist	3 (15.79)
Medical doctor	8 (42.11)
Counsellor	2 (10.53)
Telephone-based service	2 (10.53)
Self-help book	2 (10.53)
Suicidality and self-harm^b^, *n* (%)	
Past suicidality	10 (52.63)
Past suicidality in previous 12 months	4 (21.05)
Past suicidality in previous 28 days	2 (10.53)
Past self-harm	2 (10.53)
Past suicide attempt	2 (10.53)
BED severity based on DSM-5 criteria, *n* (%)	
Mild (1 to 3 weekly objective binge episodes)	10 (52.63)
Moderate (4 to 7 weekly objective binge episodes)	8 (42.11)
Severe (8 to 13 weekly objective binge episodes)	1 (5.26)

a Cultural background was based upon the classification stipulated by the Australian Bureau of Statistics (2016); the single selection item required the self-identification of a primary cultural background by participants, although there was an option to self-identify specific ethnic or cultural group/s via a free text field.

b Denotes questions that could be answered multiple times by participants.This table was originally published in Rom and colleagues (32) and has been modified to remove baseline data that now appears in Table 4. This table is part of an open access article under the terms of the Creative Commons Attribution-NonCommercial-NoDerivs License, which permits use and distribution in any medium, provided the original work is properly cited, the use is non-commercial, and no modifications or adaptations are made.

### 3.2. Treatment outcomes

The findings across primary outcomes are reported below, with all other outcomes outlined in Table 4, including 95% confidence intervals, adjusted means, and standard error.

**Table 4:** Means (M), standard error (SE), and treatment outcomes at pre-treatment, post-treatment and follow up (*N* = 19).

	*M (SE)*			Pre-post-follow up	*B* [95% *CI*]	Post-follow up
		Pre	Post		Follow up		Pre-post
EDE-Q						
OBE frequency	12.20 (1.24)	2.36 (1.26)	2.33 (1.55)	−4.94^***^ [−7.02, −2.85]	−9.84^***^ [−13.10, −6.59]	0.03 [−3.66, 3.72]
OBE days	12.09 (1.13)	2.50 (1.17)	2.00 (1.33)	−5.04^***^ [−6.83, −3.26]	−9.58^***^ [−12.45, −6.71]	0.50 [−2.61, 3.62]
Restraint	1.89 (0.40)	0.28 (0.41)	1.13 (0.50)	−0.38 [−1.01, 0.25]	−1.60^**^ [−2.62, −0.59]	−0.85 [−1.94, 0.25]
Eating concern	2.93 (0.25)	0.65 (0.27)	0.85 (0.33)	−1.04^***^ [−1.52, −0.56]	−2.28^***^ [−3.05, −1.50]	−0.20 [−1.02, 0.63]
Shape concern	4.12 (0.33)	2.36 (0.37)	2.13 (0.50)	−1.00^**^ [−1.61, −0.38]	−1.76^***^ [−2.73, −0.78]	0.24 [−0.87, 1.34]
Weight concern	3.71 (0.34)	1.91 (0.38)	1.77 (0.52)	−0.97^**^ [−1.62, −0.32]	−1.81^**^ [−2.84, −0.77]	0.14 [−1.06, 1.33]
Global score	3.16 (0.22)	1.33 (0.24)	1.44 (0.35)	−0.86^***^ [−1.32, −0.40]	−1.83^***^ [−2.52, −1.15]	−0.11 [−0.91, −0.69]
TFEQ						
Cognitive restraint	8.87 (1.09)	8.09 (1.20)	7.26 (1.50)	−0.81 [−2.45, 0.84]	−0.78 [−3.50, 1.94]	0.83 [−2.22, 3.88]
Disinhibition	14.49 (1.03)	11.21 (1.19)	11.83 (1.46)	−1.33 [−3.10, 0.45]	−3.28^*^ [−6.29, −0.27]	−0.63 [−3.81, 2.56]
Hunger	11.49 (1.00)	7.96 (1.20)	7.60 (1.38)	−1.95^*^ [−3.59, −0.30]	−3.53^*^ [−6.44, −0.63]	0.36 [−2.72, 3.45]
Global score	34.85 (2.38)	27.50 (2.76)	26.95 (3.24)	−3.95 [−7.94, 0.05]	−7.36^*^ [−14.43, −0.29]	0.54 [−6.80, 7.88]
EDQOL						
Psychological	2.60 (0.22)	1.29 (0.30)	1.43 (0.26)	−0.58^**^ [−0.95, −0.22]	−1.31^***^ [−2.04, −0.58]	−0.14 [−0.89, 0.60]
Physical/cognitive	1.05 (0.14)	0.24 (0.15)	0.45 (0.17)	−0.30^**^ [−0.51, −0.10]	−0.82^***^ [−1.14, −0.49]	−0.21 [−0.58, 0.16]
Financial	0.58 (0.08)	0.10 (0.09)	0.09 (0.09)	−0.24^***^ [−0.36, −0.12]	−0.48^***^ [−0.70, −0.26]	0.01 [−0.21, 0.23]
Work/school	0.31 (0.05)	0.05 (0.06)	0.01 (0.06)	−0.15^***^ [−0.23, −0.07]	−0.26^**^ [−0.43, −0.10]	0.04 [−0.13, 0.21]
Global score	1.37 (0.11)	0.53 (0.14)	0.64 (0.14)	−0.36^***^ [−0.55, −0.17]	−0.84^***^ [−1.18, −0.49]	−0.12 [−0.44, 0.21]
Other outcomes						
K10 total score	20.42 (1.12)	15.17 (1.17)	16.34 (1.51)	−2.04^*^ [−3.82, −0.26]	−5.24^***^ [−7.96, −2.52]	−1.16 [−4.34, 2.02]
BMI	32.79 (1.57)	32.74 (1.62)	32.72 (1.69)	−0.03 [−0.83, 0.76]	−0.06 [−1.27, 1.16]	0.01 [−1.30, 1.32]

OBE, objective binge episode; SBE, subjective binge episode days; CI, confidence intervals; EDE-Q, Eating disorder examination questionnaire; TFEQ, The three-factor eating questionnaire; EDQOL, The eating disorder quality of life questionnaire; K10, Kessler psychological distress scale; BMI, Body mass index. Means and standard error were calculated from the model.

* *p* < 0.05;

** *p* < 0.01;

*** *p* < 0.001.

With regards to objective binge episode (OBE) frequency, there was a significant decrease across all three timepoints (*b* = −4.94, *p* < 0.001). Between pre-and post-treatment there was also a significant decrease (*b* = −9.84, *p* < 0.001) in OBE frequency and this was a large effect (*g* = 1.03, 95% CI [0.69, 1.37]). There was no significant difference between post-treatment and follow up (*b* = 0.03, *p* = 0.99, *g* = 0.003).

Similarly, there was a significant decrease in the EDE-Q global score across all three timepoints (*b* = −0.86, *p* < 0.001). Between pre-and post-treatment there was also a significant decrease in the EDE-Q global score (*b* = −1.83, *p* < 0.001) and this was a large effect (*g* = 1.62, 95% CI [1.02, 2.23]). There was no significant difference between post-treatment and follow up (*b* = −0.11, *p* = 0.78, *g* = 0.10).

The relative change in descriptive indices of clinical significance was calculated between pre-and post-treatment, and post-treatment and follow up based on the pre-treatment sample (*n* = 19). The number of participants who were abstinent from binge-eating (in the previous month) increased by 11% (*n* = 2) at post-treatment and a further 5 % (*n* = 1) at follow-up. Participants who met the criteria for recovery from binge-eating (<4 OBEs in the previous month) increased by 42% (*n* = 8) at post-treatment, which was sustained at follow up. Furthermore, participants who met the criteria for ‘good outcome’ (i.e., an EDE-Q global score < 2.77) increased by 32% (*n* = 6) at post-treatment and decreased by 5% (*n* = 1) at follow up. Table 5 outlines results at pre-treatment, post-treatment, and follow up.

**Table 5:** Descriptive indices of clinical significance at pre-treatment, post-treatment and follow up (*N* = 19).

	*n (%)*			Pre	Post	Follow up
Abstinence from binge-eating in previous month	1 (5)	3 (16)	4 (21)
Recovery from binge-eating (i.e., <4 OBEs in the previous month)	2 (11)	10 (53)	10 (53)
Good outcome (i.e., an EDE-Q global score < 2.77 in previous month)	7 (37)	13 (68)	12 (63)
Missing participant data	--	5 (26)	6 (32)

#### 3.2.1. Weekly outcomes

**Figure 3:**
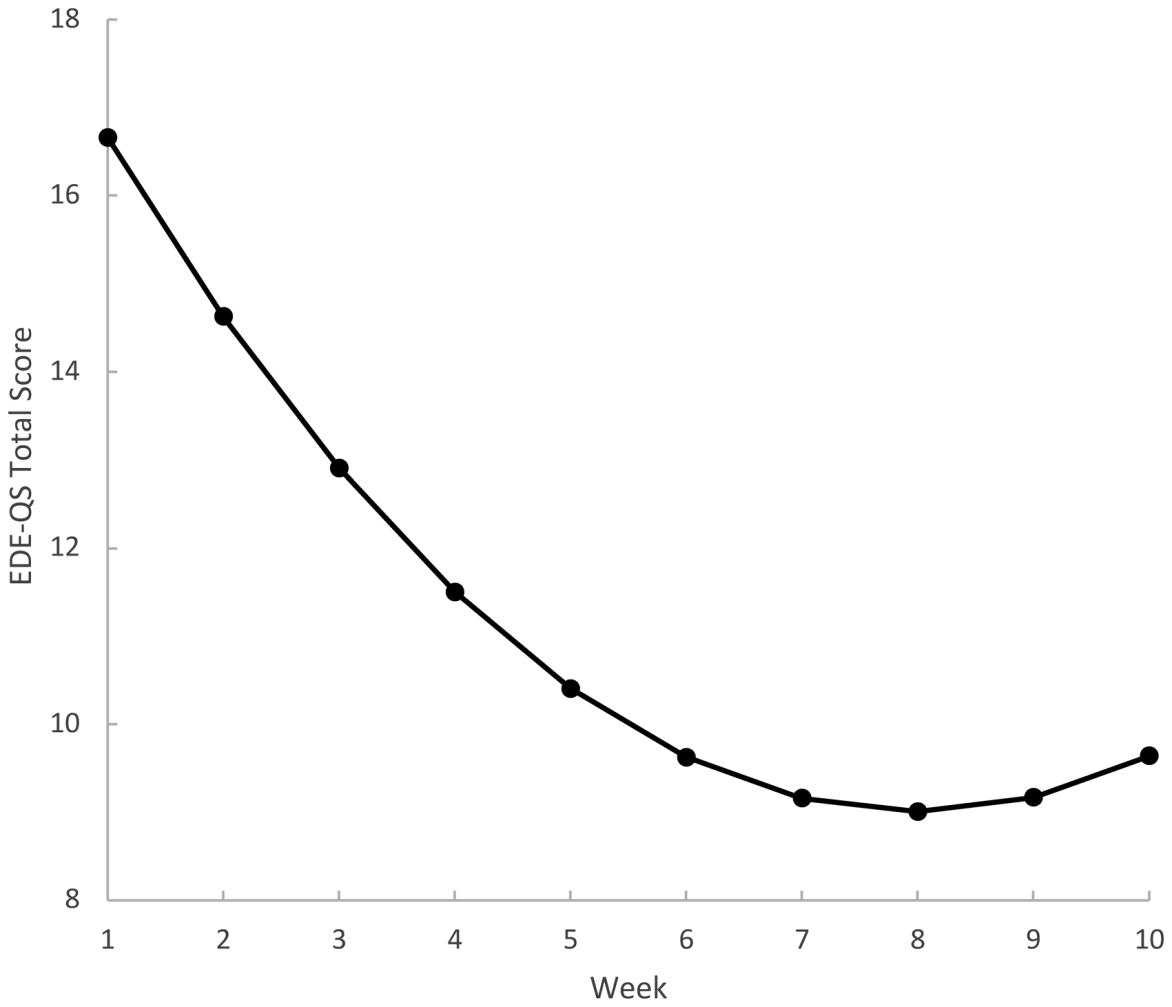
Model means of weekly eating disorder examination - questionnaire short (EDE-QS) – total scores. Weekly scores reflect reference timeframe of previous 7 days. Week 1 and week 5 represent the pre-post timepoints for the previous evaluation in participants after four sessions of the eTherapy.

**Figure 4:**
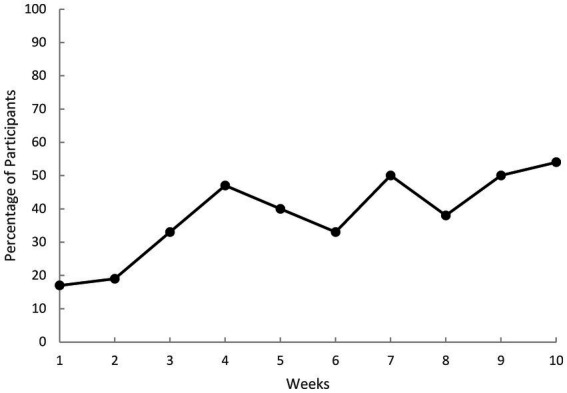
Percentage of participants abstinent from binge-eating each week as per the eating disorder examination - short (EDE-QS). Weekly scores reflect reference timeframe of previous 7 days. Binge-eating abstinent was defined as zero objective binge episode days in the previous 7 days.

Across the 10-weekly questionnaires, time (*b* = −2.50, *p* < 0.001) and time squared (*b* = 0.16, *p* < 0.001) were statistically significant, indicating a trend of decreasing EDE-QS total scores. Figure 3 displays the time squared model across weekly EDE-QS total scores, illustrating a marked slowing in the rate of change between week five and six, which appears to plateau at week seven. Figure 4 illustrates the percentage of participants who achieved zero OBE days in the previous week at these same timepoints. In the last four weekly timepoints, the percentage of participants with zero OBE days (in the previous week) ranged between 38% (*n* = 5) and 54% (*n* = 7).

### 3.3. Feasibility

#### 3.3.1. Program dropout and adherence

Out of 19 participants, an overall study dropout rate of 26% was observed (*n* = 5). Two participants voluntarily withdrew: one cited changed availability before commencing the program and the other withdrew due to co-occurring mental health concerns after completing module one. A further two participants did not adhere per-protocol (i.e., did not attend three consecutive support sessions) and were considered disengaged after completion of module one and module two. In addition, one participant was discontinued from the study due to the emergence of secondary symptoms which required more intensive support.

Adherence indices were evaluated for participants who completed a minimum of one module of eTherapy content (*n* = 18). On average, participants completed 84% of module content (*M* = 8.39, *SD* = 3.33) and attended 86% of the support sessions (*M* = 8.56, *SD* = 3.07). Medians and interquartile ranges (IQR) were employed for self-monitoring data given its skewed nature. The median value of total program logins across the entire intervention period was 157 (93–234). The following median (IQR) values were found for self-monitoring entries: 346 (198–383) for food monitoring; 9 (1–26) for thought monitoring; 33 (13–60) for behaviour monitoring; 9 (0–22) for food planning; 4 (2–12) for goal setting; and 1 (0–18) for exposure challenges. The median value of self-monitoring entries per login was 6 (2–10).

#### 3.3.2. Support sessions

The duration support sessions ranged from 18.00 to 105.00 min (*M* = 37.92, *SD* = 13.17), with support clinicians spending between 5.00 to 60.00 min preparing for the session (*M* = 11.29, *SD* = 5.72). Means and standard deviations were calculated across all sessions.

### 3.4. Acceptability

Acceptability was evaluated in participants who completed the post-treatment questionnaire (*n* = 14). Ninety-three percent (*n* = 13) of participants were “extremely satisfied” with the program overall. Seventy-nine percent (*n* = 11) of participants “agree[d]” that their eating disorder thoughts had been reduced by the program, whilst 86% (*n* = 12) “strongly agree[d]” that the program had reduced their eating disorder behaviours. All participants (*n* = 14) reported that clinician support was “extremely helpful,” with 79% (*n* = 11) reporting a benefit of increased program engagement. Sixty-four percent (*n* = 9) of participants reported that there were no unhelpful aspects of clinician support, with 29% (*n* = 4) reporting worry regarding correct program completion when reporting to their support clinician. When asked which program skills were the most helpful, regular eating via the three rule was the most selected option (93%; *n* = 13), followed by urge surfing (79%; *n* = 11) and thought challenging and self-monitoring, both 71% (*n* = 10). When asked to select which components of the eTherapy were the most helpful, the self-monitoring tools were the most selected option (86%; *n* = 12), followed by the interactive activities and quizzes within sessions (57%; *n* = 8).

### 3.5. Adverse events

There were no known unexpected adverse events indicated via weekly questionnaires that evaluated suicidality/self-harm and negative affect, or via post-treatment feedback regarding the quality of the intervention. In addition, assessment of negative affect/psychological distress at pre-treatment, post-treatment and follow-up demonstrated significant reductions at post-treatment, that were maintained at follow up. Furthermore, there were no qualitative reports to clinicians during support sessions or other reports to researchers.

## 4. Discussion

The aim of this study was to investigate the acceptability, feasibility, and preliminary efficacy of a supported eTherapy intervention for people with BED or subthreshold BED. In support of the hypotheses, statistically and clinically significantly reductions in objective binge episode (OBE) frequency and the EDE-Q global score were found post-intervention and were maintained at follow up. There were also significant reductions across secondary outcomes, including additional measures of eating disorder psychopathology, quality of life, and psychological distress. Furthermore, a marked slowing in the rate of change in ED psychopathology was observed between the fifth and sixth weekly questionnaire. Participant satisfaction with the eTherapy was high, as was adherence to program content, indicating that a low intensity CBT-based online therapy, supported by non-expert clinicians in more time-limited telehealth sessions, is feasible, acceptable, and can be effectively delivered to individuals with BED.

### 4.1. Preliminary efficacy

#### 4.1.1. Primary outcomes

There was a large significant reduction in OBE frequency from pre-to post-treatment (*g* = 1.03), with this decrease maintained at 3 months follow up. This large effect exceeded the average moderate pooled effect (*d* = −0.77) observed in a meta-analysis of BED eTherapy programs (31). Although this comparison requires caution given the pooled effect occurred in studies comparing treatment to a control group, promisingly, this reduction also appeared to be clinical significant as per the following indices. Overall, average OBE frequency at follow up (2.33) represented an 81% reduction from the pre-treatment average (12.20), whilst abstinence from binge-eating at post-treatment (16%) and follow up (21%) was comparable to the range of abstinence rates (14.6–25.1%) in existing studies evaluating BED eTherapies (31). In addition, average post (2.36) and follow up (2.33) OBE frequency were well below Wagner and colleagues (29) index for recovery from BED (<4 OBEs).

Similarly, there was a statistically and clinically significant reduction from pre-to post-treatment in the EDE-Q global score, representing a large effect (*g* = 1.62) which compared well to the moderate pooled reductions (*d* = −0.77) observed in a BED targeted eTherapy meta-analysis (31). This decrease was maintained at follow up, with average post-treatment (1.33) and follow up (1.44) scores below the population norm (1.52) for Australian women (47).

#### 4.1.2. Secondary outcomes

All EDE-Q subscales (excluding dietary restraint) demonstrated significant reductions across timepoints, with post-treatment and follow up scores reduced to sub-clinical levels as per population norms (47). The non-significance of dietary restraint is consistent with research suggesting its reduced level in those with BED (2) and was further reflected in the average pre-treatment dietary restraint score (1.89) which was within one standard deviation of the population norm. There were also significant reductions across timepoints in the EDQOL total score (i.e., illness related quality of life) and subscales, with all post and follow up scores reduced to below, or less than one standard deviation above, population norms of women not experiencing an eating disorder (46). In addition, significant reductions across timepoints in the K10 total score (i.e., psychological distress) represented a clinically significant shift from an average score representative of a ‘mild mental disorder’ (i.e., a pre-treatment score of 20.42) to one suggesting ‘likely to be well’ (i.e., post-treatment and follow up scores of 15.17 and 16.34, respectively) as per descriptive cut-offs used in general practice (51). These clinically significant improvements in quality of life and psychological distress are promising given the importance of ED interventions to promote positive outcomes across other domains, and not solely eating disorder cognitions and behaviours (52). Consistent with convergent findings across CBT-based programs for BED (4), there was no significant differences in BMI across timepoints, further supporting that weight loss or gain is not an expected treatment outcome in CBT-based treatment for BED. Overall, there was no noteworthy clinically significant change in TFEQ scores.

#### 4.1.3. Rate of weekly change

Weekly means from the modelling indicated a statistically significant decrease in the total EDE-QS scores (i.e., ED psychopathology) across the 10-weekly timepoints. There was a substantial slowing of the rate of change observed between the fifth and six weekly questionnaire (i.e., after four eTherapy sessions), which adds important context to the previous evaluation completed after session four in this sample (32). A marked slowing in the rate of change at this juncture further supports the theory that behavioural techniques introduced in the preliminary sessions of CBT treatment (i.e., weekly weighing, self-monitoring of food intake, regular eating) may facilitate early and rapid symptom change (34, 53, 54). Alternatively, it may be that participants respond better in earlier treatment stages, with diminishing returns over time. Both assertions would have important implications for both the ordering of therapeutic elements within programs and the determination of appropriate program durations and require further investigation. For example, although a brief, four-session eTherapy program may substantially reduce ED psychopathology in BED, programs of increased duration may be needed to embed learning and maintain gains over time. Longer programs may also be better suited for those with more severe symptomatology and a longer duration of illness due to a higher symptom burden (4).

### 4.2. Acceptability

Acceptability for the eTherapy was high, with the majority of participants (93%) reporting the highest possible level of satisfaction with the program, and all participants indicating that clinician support was extremely helpful in completing the program. This reflects the amenability of those with an ED to treatment via digital programs, particularly when there is a level of support provided by a clinician (16). Regular eating was identified by the majority of participants (93%) as the most helpful skill, and the self-monitoring tools the most helpful component (86%), of the eTherapy program. This is noteworthy given the sub-clinical level of dietary restraint in this sample but aligns with evidence suggesting regular eating may be the most potent mechanism behind reductions in OBEs in CBT-based interventions in transdiagnostic non-underweight ED samples (53, 55).

### 4.3. Feasibility

High rates of adherence were found across program content (i.e., 84% of content was completed and 86% of support sessions were attended), which exceeded the average content adherence rate of 50% found in similar supported internet-based mental health interventions (56). The dropout rate (26%) was lower than the average rate of 32% estimated in a meta-analysis of BED eTherapies (57) and comparable to the rates of 21 and 16% found in the pilots of the briefer, four-session version of this eTherapy in individuals with BED and BN (32, 54). The majority of those who dropped out after commencing content (*n* = 3) did so early in the program (module one and two), with two participants indicating prior co-occurring psychological symptoms. This aligns with evidence suggesting early dropout in ED-focused online programs might be predicted by baseline characteristics that reflect more complex mental health presentations (58), affirming the need for additional support for such individuals, e.g., face-to-face CBT (59). Overall, the significant change found across key outcome measures, in addition to high levels of adherence and lower than average dropout, support the feasibility of this eTherapy program.

### 4.4. Strengths, limitations, and future research

Some of this study’s key strengths include a recruited clinical sample that met DSM-5 diagnostic criteria for BED via an interview (1). This is noteworthy given the identified need for further eTherapy evaluation in purely BED populations (31). Furthermore, the predominantly mild to moderate binge-eating symptomatology identified in this sample reflects recommendations on the use of eTherapy programs in individuals with this severity of symptomatology as per a stepped care approach to treatment (21). Support sessions with participants were primarily facilitated by trained research assistants, contrasting with previous studies that have employed clinical psychologists in this role (28, 29, 60). Given the aspiration of eTherapies to address issues of accessibility, scalability, and cost (11, 23, 24), this study’s use of other professionals in this role, whilst achieving similar results, supports this utility of BED-targeted eTherapies within a stepped-care model. Overall, these features, in addition to the naturalistic setting of this study (i.e., participants completing the intervention and support sessions via digital devices in their homes), approximates a community implementation and thus results may reflect what would be observed in everyday application.

This study, however, has some limitations. Although findings demonstrated consistent, significant reductions across all variables of interest, the lack of control group and small, women-only sample, limits causal inference and results should be considered provisional until confirmatory replication in an RCT with a more diverse and appropriately sized sample. Furthermore, a longer follow up timepoint (i.e., 1 year) would assist in ascertaining longer-term treatment efficacy. In addition, although no known unexpected adverse effects to the intervention were indicated via weekly monitoring in questionnaires evaluating suicidality and negative affect (or reported to support clinicians in weekly sessions), the absence of specific qualitative questioning regarding adverse effects may limit understanding, particularly with respect to those who dropped out.

Overall, findings from this study provide preliminary support for the acceptability, feasibility, and efficacy of a supported BED-targeted eTherapy treatment program. The program was well received by most participants, demonstrating strong feasibility in high levels of adherence to program content, and preliminary efficacy via large-sized, clinically significant reductions in primary outcomes (OBE frequency and ED psychopathology) post-treatment, which were maintained at follow up. Observations of weekly ED psychopathology across the duration of the eTherapy suggest a marked slowing in the rate of change in CBT for BED after session four, which appears to further plateau as therapy continues. These findings contribute to a small but emerging evidence-base of BED eTherapies that suggests their possible efficacy in reducing binge-symptomatology and ED psychopathology, and potentially over comparatively short time frames. Future research should replicate findings in an appropriately sized RCT and further evaluate more individualised eTherapy interventions that focus on the most potent or individually relevant therapeutic elements and further investigate optimal treatment durations. Given the barriers that maintain unacceptably low levels of treatment uptake, eTherapy programs present a significant opportunity in the provision of much needed, accessible evidence-based treatment for those with BED.

## Data availability statement

The original contributions presented in the study are included in the article/supplementary materials, further inquiries can be directed to the corresponding author.

## Ethics statement

The studies involving humans were approved by Sydney Local Health District RPA Research Ethics and Governance Office. The studies were conducted in accordance with the local legislation and institutional requirements. The participants provided their written informed consent to participate in this study.

## Author contributions

SM, SB, and SR conceived the research methodology and conducted the investigation. SM, MF-T, and JM-W supervised the project. SR and PA administered the project, including the intervention. MK and SR conducted the formal analysis. SR wrote the original draft. SM, SB, JM-W, PA, MK, and MF-T reviewed and edited the paper. All authors contributed to the article and approved the submitted version.
